# A Novel Plate-Based System (UNIMAX) for Posterior Instrumented Spinal Fusion

**DOI:** 10.7759/cureus.11080

**Published:** 2020-10-21

**Authors:** Madhavan Pisharodi, Zaid Aljuboori, Vijay K Goel, Haring J Nauta

**Affiliations:** 1 Neurosurgery, South Texas Neurosurgery Associates, Brownsville, USA; 2 Neurosurgery, University of Louisville School of Medicine, Louisville, USA; 3 Bioengineering, University of Toledo, Toledo, USA

**Keywords:** spinal fusion, instrumentation, scoliosis, postero-lateral fusion, titanium, plates

## Abstract

Introduction

The polyaxial head pedicle screw-rod system is a commonly used spinal instrumentation technique to achieve stabilization, deformity correction, and bony fusion. We present a novel plate-based pedicle screw system (UNIMAX^TM^) that can be used for multi-level instrumentation with potential advantages for selected applications.

Methods

Bilateral titanium monoaxial pedicle screws are linked at each level by robust transversely oriented cross plates forming ring constructs capable of rigid triangulation at each level. The cross plates are then interconnected by sagittally oriented rigid plates situated medial to the screw heads. Biomechanically, the construct was tested for quasi-static torsion and fatigue in axial compression. The system is approved by the Food and Drug Administration (FDA). The system and case examples are presented showing its potential advantages.

Results

The quasi-static torsion, the mean for the angular displacement, torsional stiffness, and torsional ultimate strength was 2.5 degrees (SD ± 0.8), 5.3 N-m/mm (SD ± 0.7), and 21.6 N-m (SD ± 4.4). For the fatigue in axial compression, the closed ring construct failed when the applied load and bending moment were ≥ 1500 N and ≥ 60 N.m. This system maximizes the construct rigidity, allows easy extension to adjacent levels, and can be incorporated intuitively into practice. The ring construct with triangulation at each level, together with its biomechanical robustness, predicts a high pullout resistance. It requires an open posterior approach incompatible with minimally invasive techniques.

Conclusion

This system may have advantages over the screw-rod systems in carefully selected situations requiring extra rigidity and high pull-out strength for complex reconstructions, sagittal and/or coronal correction, patients with poor bone quality, revisions, and/or extension to adjacent levels.

## Introduction

Instrumented spinal fusion is a procedure that uses metal implants to minimize or eliminate the movement of the spinal column to promote bony fusion at the implanted levels [1]. The inception of the Harrington rod instrumentation and its successful use to treat scoliosis was a major advancement in spinal surgery [2]. However, it lacked the versatility needed to manage different spinal pathologies, including the common degenerative conditions. Pedicle screw stabilization with plates was introduced in 1970 by Roy Camille and provided excellent rigidity but was practical for only one level fusion because of the difficulty in adapting the plates to multiple levels [3]. Monoaxial pedicle screw systems with bendable rods provided a major advancement by allowing the interconnection of screws at multiple levels thus enabling the treatment of a multitude of spinal conditions [1,4]. The addition of the polyaxial screw head added further practicality to the construct and allowed adaptation to minimally invasive techniques [1,4].

Notwithstanding the popularity of the polyaxial head screw-rod systems now in use, they have several drawbacks, including a less than optimal rigidity and a reduced bony surface available for fusion, which might necessitate the inclusion of additional spinal levels or anterior interbody fusion in the final construct. The screw-rod constructs have an appreciable failure rate in patients with a poor bony quality or multiple revisions [5-9]. Construct revision or extension for an adjacent-level disease is another disadvantage of the screw-rod systems because they typically require the removal of the rods with some fused bone. Collectively, these shortcomings signaled the need for an innovative design striving for improved biomechanical characteristics that might achieve a higher rate of posterolateral bony fusion under particularly demanding situations, especially those where the screw-rod systems are lacking. Here, we present a biomechanical evaluation and clinical experiences with a novel titanium plate-based pedicle screw system (UNIMAX) for posterior instrumented spinal fusion. It appears that this plate-based system, with its rigid triangulation at each level, may have several potential advantages over currently popular screw-rod systems in carefully chosen clinical settings.

## Materials and methods

**Figure 1:**
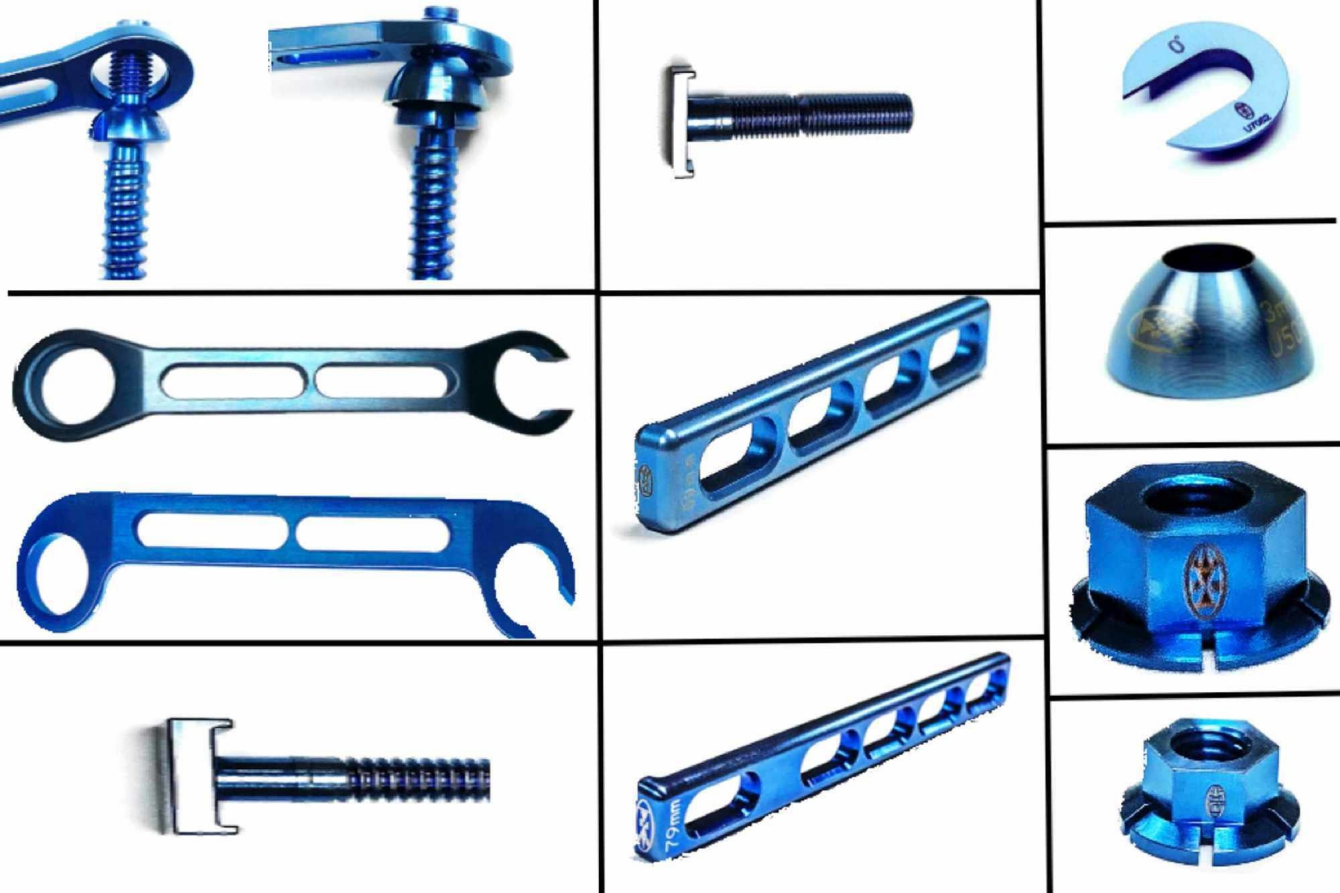
A picture that shows the different components of the UNIMAX system.

The system is titanium-based and was cleared by the Food and Drug Administration (FDA) 510(k) (No. K024313). It includes monoaxial pedicle screws, horizontal and vertical plates, and a multitude of washers, nuts, and bolts to connect these components (Figure 1). 

**Figure 2:**
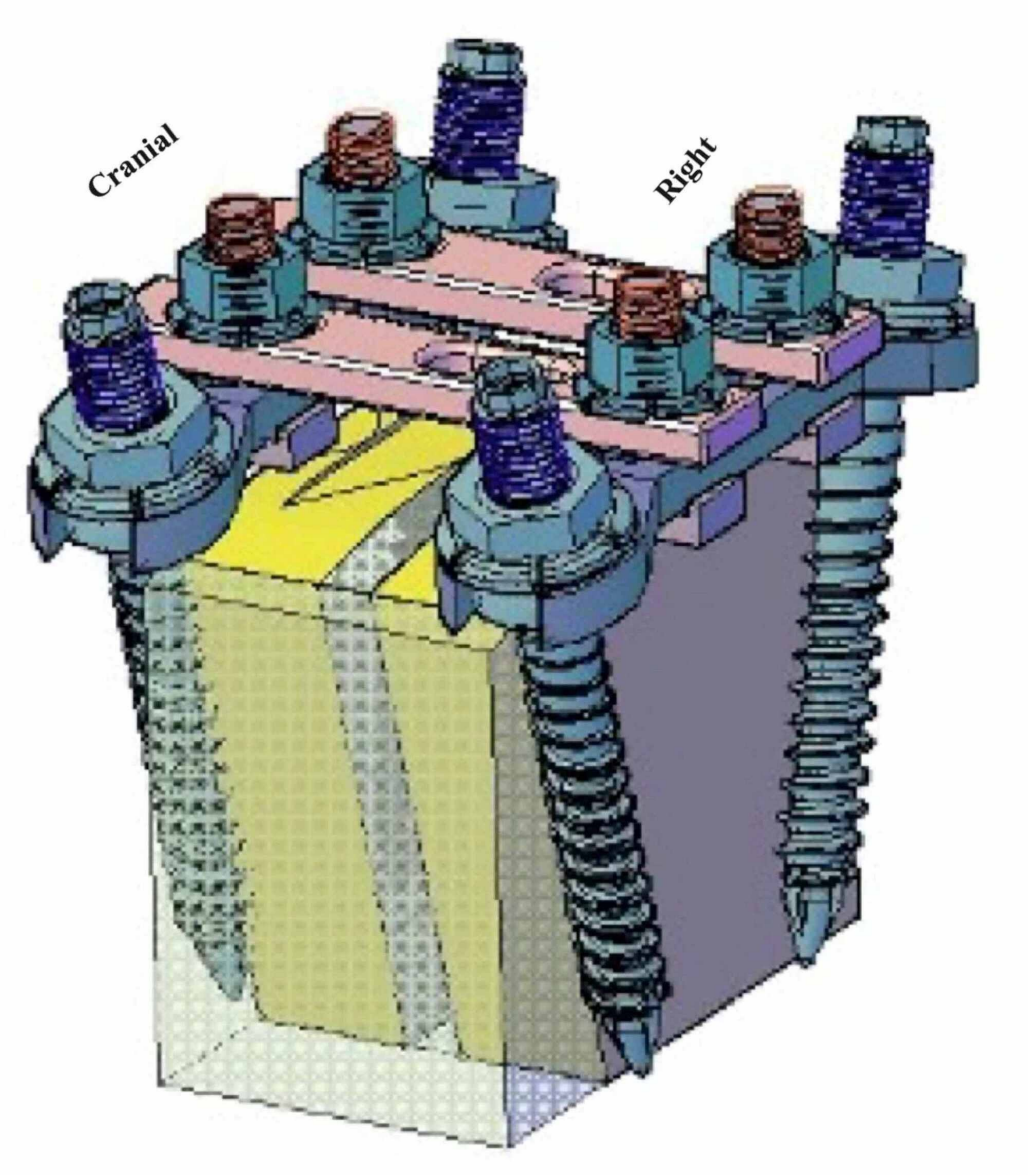
An art sketch depicts the configuration of the UNIMAX system after placement Note the robust triangulation designed to maximize pull-out strength. The cross plates at each segmental level are in blue and the sagittally oriented plates are in pink. The sagittal plates can be contoured and stacked for extra rigidity as needed.

The concept behind the design is to create a ring construct forming rigid triangulation at each spinal level. This is done by interconnecting the screw heads at each level with a robust transversely oriented cross plate. The cross plates are then interconnected by sagittally oriented rigid plates situated medial to the screw heads (Figure 2). These sagittal plates can be contoured and stacked to achieve the desired lordosis and for additional rigidity as needed. A detailed technical description - the UNIMAX product catalog - can be found here (https://www.paramountsurgicals.com/pdf/paramountsurgicals_catalog.pdf).

Biomechanical testing

The construct was tested for quasi-static torsion, fatigue in axial compression, and quasi-static axial compression and bending. 

Operative technique

**Figure 3:**
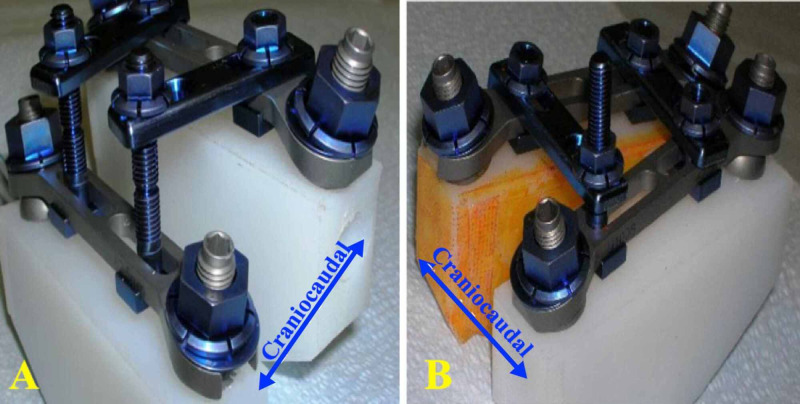
A model that shows the various stages of placement of the UNIMAX system (A) shows the use of long square bolts to correct spondylolisthesis. The extra length of the square bolts is broken off. (B) shows the correction of rotoscoliosis when the vertical plate is forced onto the horizontal plate. By tightening the nut of the long square bolt.

**Figure 4:**
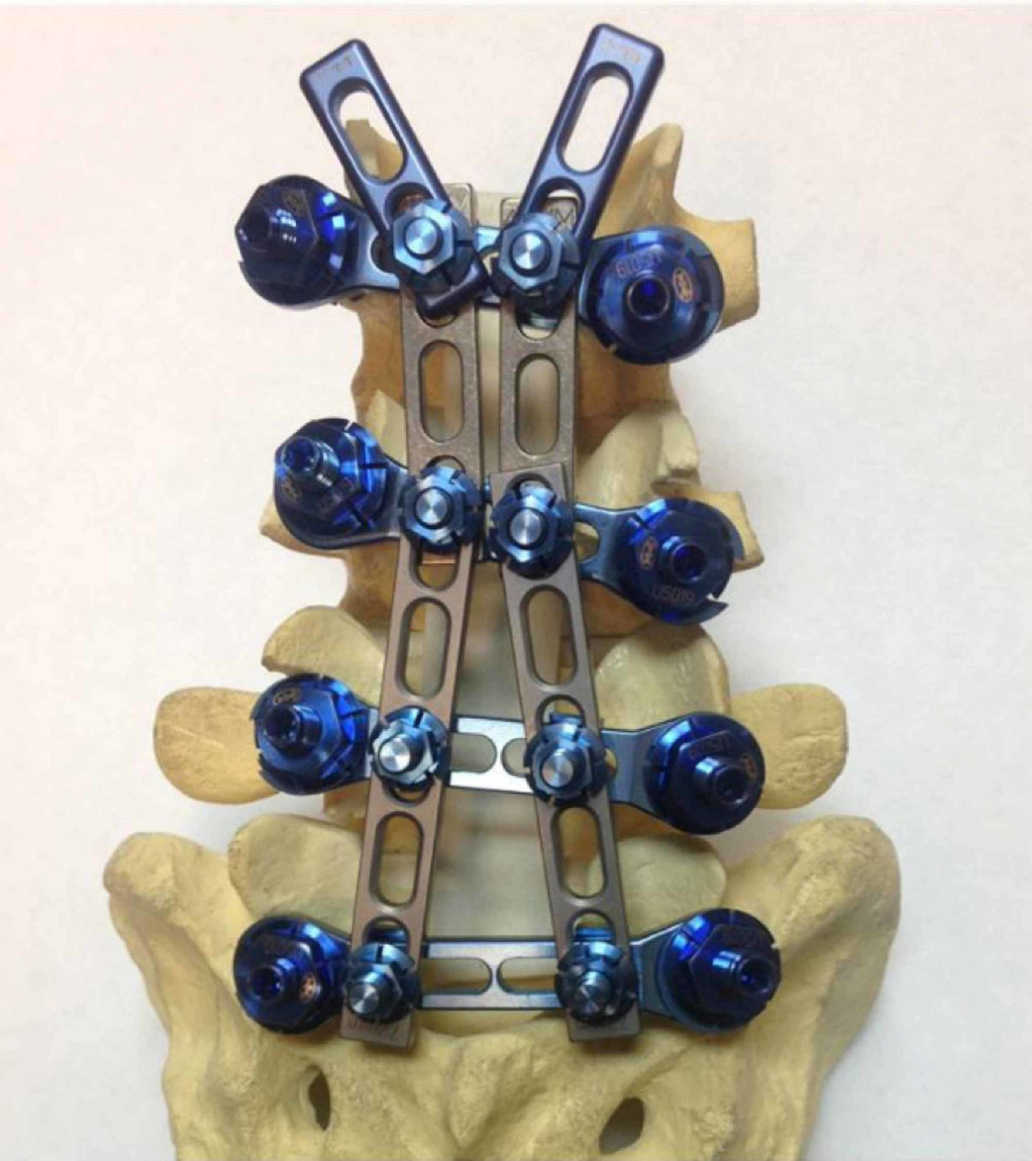
A lumbosacral spine model with the UNIMAX system in place, from L3 to S1, and the ability to add L2/3 without removing L3/S1

The system requires a standard open operative exposure, which includes a midline skin incision followed by subperiosteal dissection of the paraspinal muscles to expose the spinous processes, laminae, facets, and transverse processes. Then, a full laminectomy is required at each level to allow enough space for the construct placement. In cases requiring deformity correction, a facetectomy is typically performed as well. The pedicle screws are inserted using a standard technique [10]. Horizontal cross plates preloaded with square bolts are then applied onto the pedicle screw heads followed by placement of the flat washers and the large flange nuts, initially attached loosely to allow for later adjustment of the plates as needed to wed optimally. The vertical plates are then prepared (bent as needed), attached paramedian on each side to each horizontal cross plate, and linked with the square bolts to achieve the desired amount of sagittal and or coronal correction. The small flange nuts are tightened first, and only then are the large flange nuts (Figures 3-4) on the pedicle screws tightened fully. Additional steps to promote bony fusion can be used as needed, including the application of the autologous harvested bone, osteoinductive factors, osteoconductive matrices, and/or osteogenic factors.

Case illustration

Case 1

**Figure 5:**
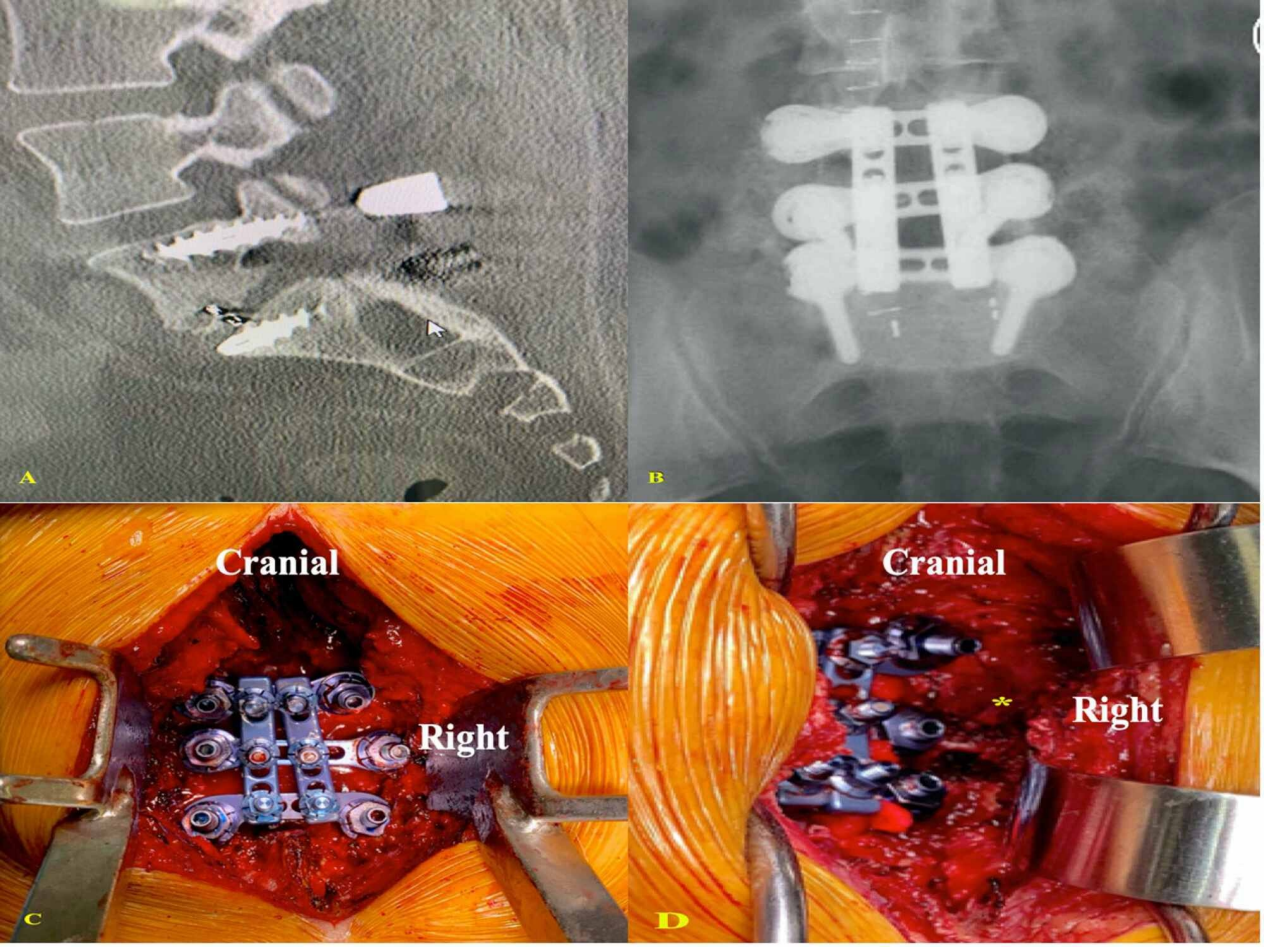
(A) CT lumbar spine (sagittal view) shows evidence of construct failure. (B) X-ray (AP view) shows the UNIMAX system after revision. (C) Intraoperative picture shows the UNIMAX system after placement. (D) Intraoperative picture shows the instrumentation-free large bony surface area (*) in the lateral region, which promotes fusion AP: anteroposterior

A patient presented with back pain because of pseudarthrosis after a lumbar fusion using the pedicle screw-rod system and interbody cage (Figure 5A). The imaging showed subsidence of the interbody device. The patient underwent decompression with a revision of the construct with the UNIMAX system. The patient symptoms resolved after surgery and the procedure resulted in adequate posterior instrumented fusion without the need to revise the interbody cage, which carries significant morbidity (Figures 5B-5D). 

Case 2

**Figure 6:**
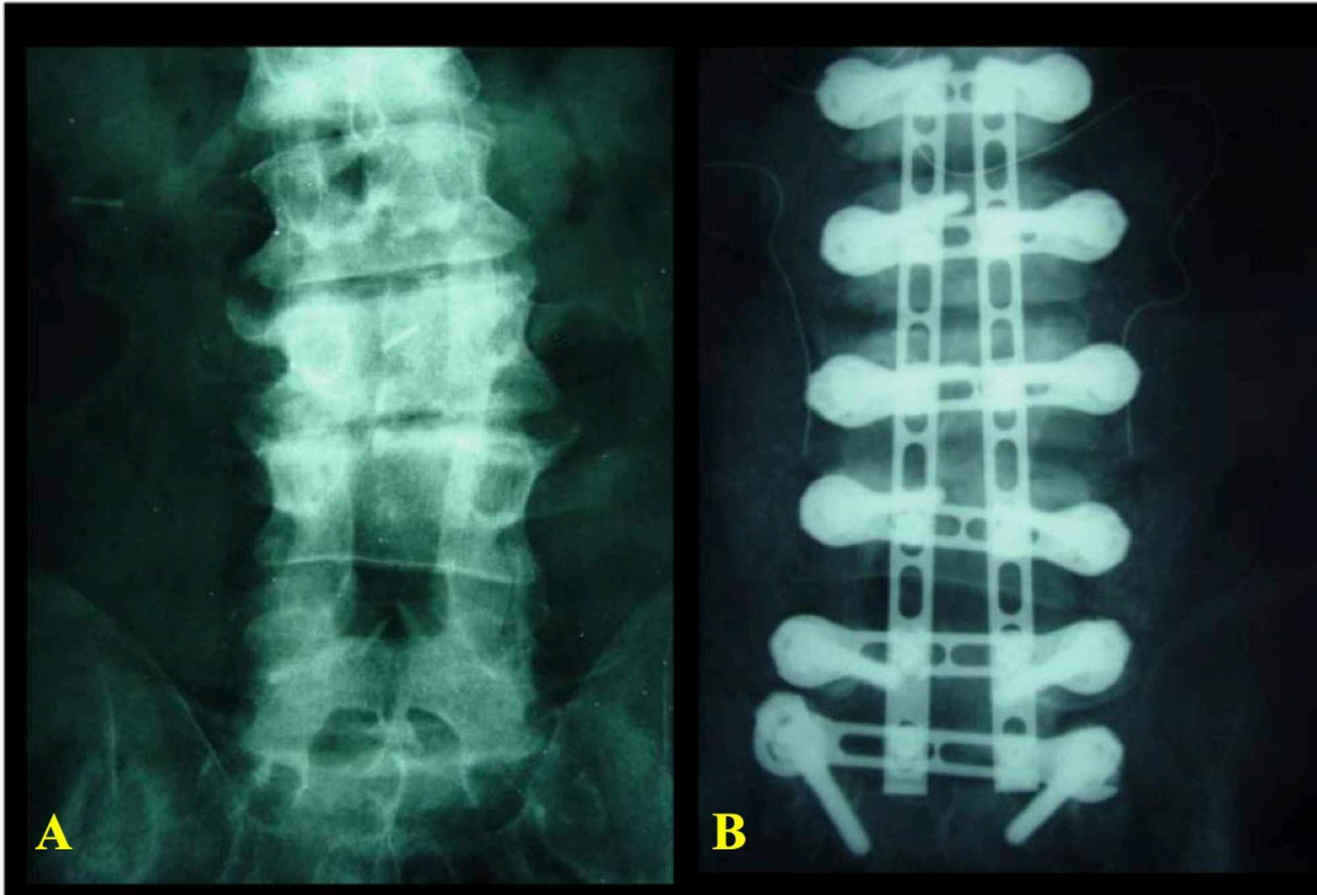
X-ray of the lumbar spine (AP view) that shows (A) levoscoliosis of the lumbar spine, (B) the lumbar spine after the correction of scoliosis with the UNIMAX system

A patient presented with lumbar coronal deformity (levoscoliosis) (Figure 6A), who underwent a correction using the UNIMAX system modular design. After facetectomy and the placement of screws and horizontal plates (ring construct), each vertebra behaved like a separate, adjustable unit. The correction was achieved by applying distraction on concavity and compression on the convexity of curvature (Figure 6B).

Case 3

**Figure 7:**
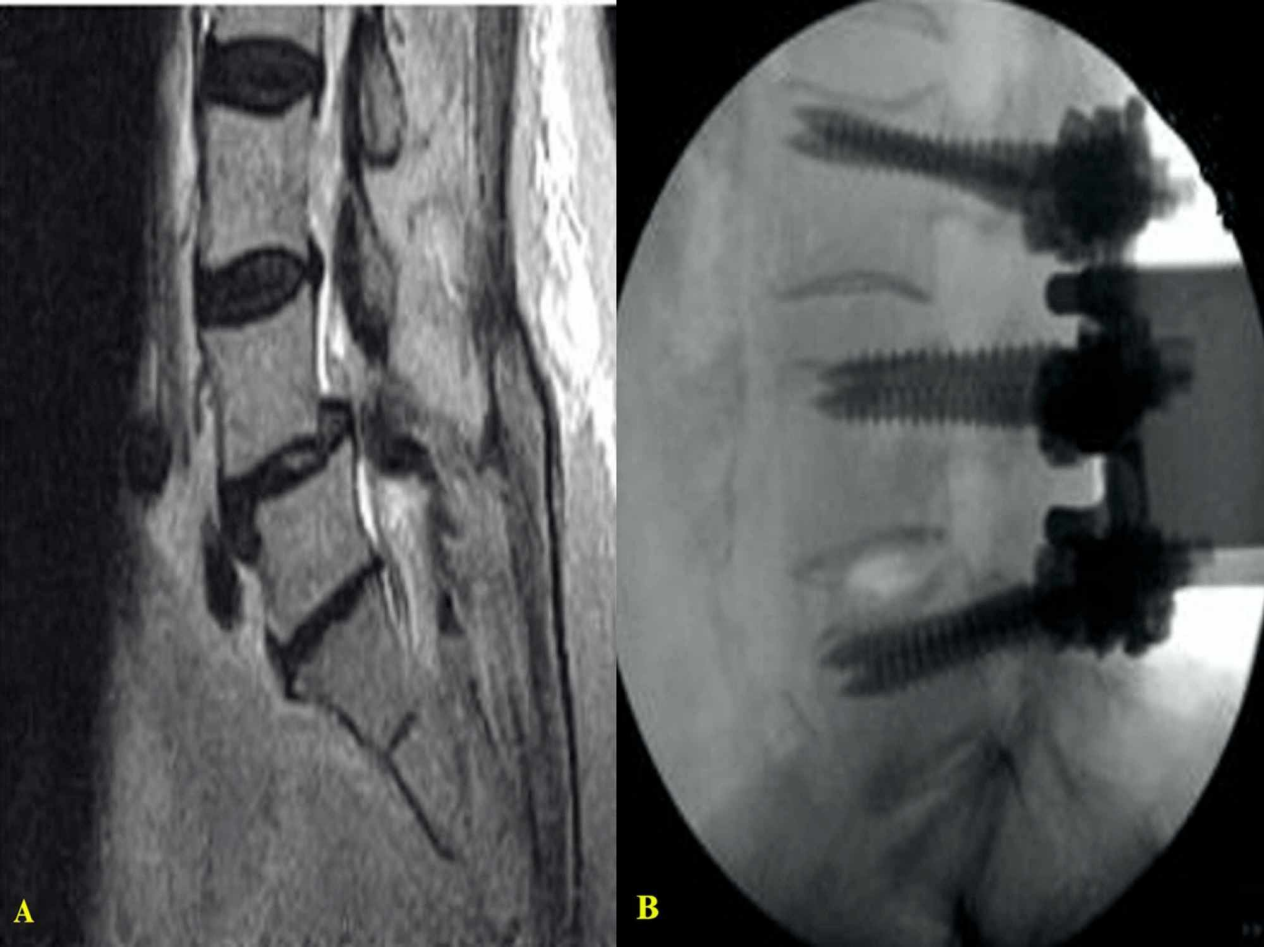
(A) CT lumbar spine (sagittal view) shows severe spinal stenosis due to L4-5 anterolisthesis. (B) Intraoperative X-ray (lateral view) shows the correction achieved with the UNIMAX pedicle screw-plate system

A patient presented with incomplete cauda equina syndrome. Imaging showed L4-5 anterolisthesis with severe canal stenosis (Figure 7A). The patient underwent a lumbar posterior decompression (no discectomy was performed) with a reduction and instrumented fusion of the affected level using the UNIMAX system (Figure 7B). Postoperatively, the patient’s symptoms resolved.

Case 4

**Figure 8:**
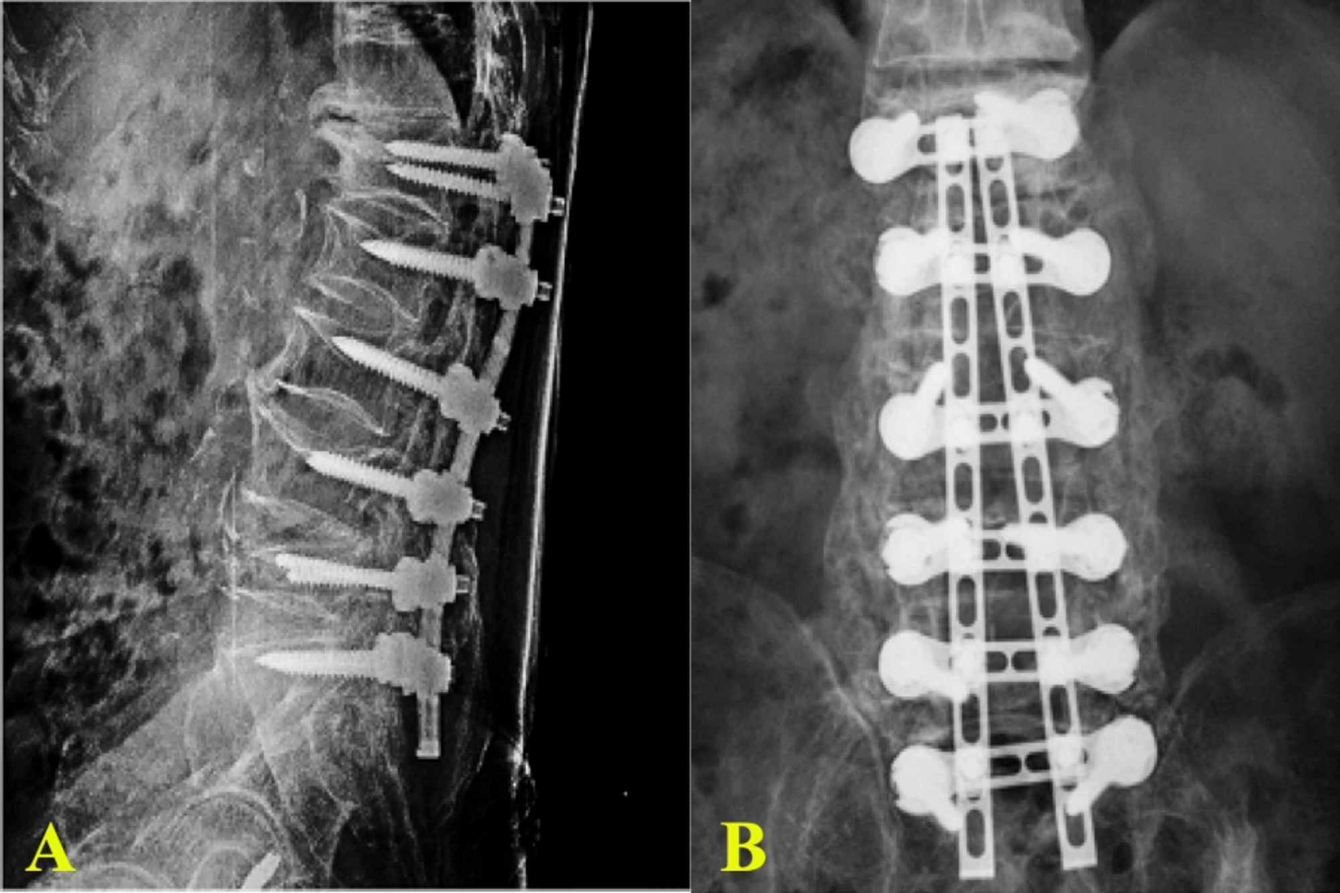
Postoperative X-ray images (AP view (A), lateral view (B)) that show lumbar compression fractures and the instrumented fusion of the L1-S1 levels using the UNIMAX system

A patient presented with multiple lumbar spine compression fractures because of osteoporosis. The UNIMAX system was used for stabilization and posterolateral fusion (Figures 8A-8B].

Case 5

**Figure 9:**
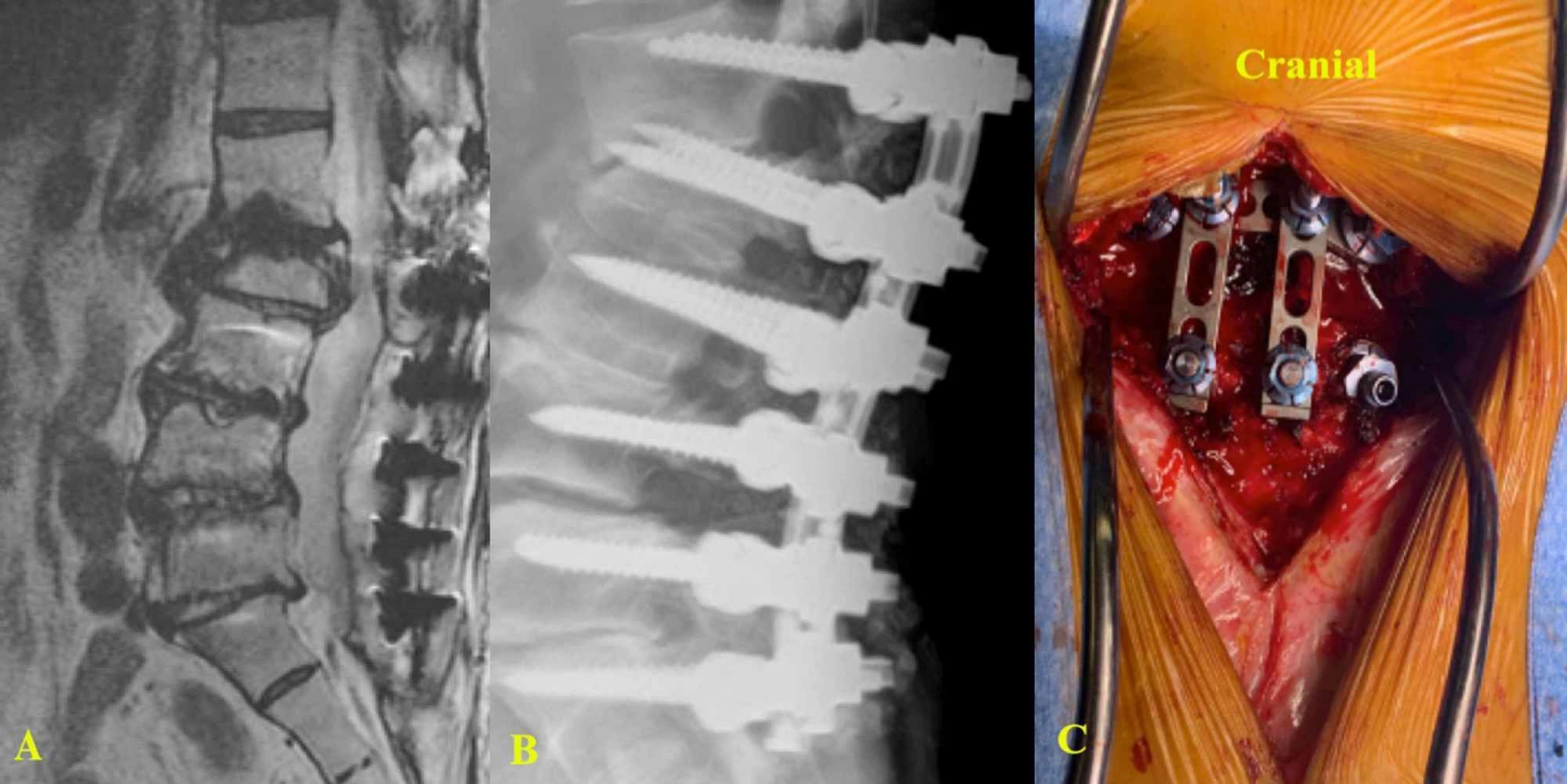
Images of a patient with a previous L2-S1 posterior fusion using the UNIMAX system (A) MRI lumbar spine (sagittal view) shows a new L2 vertebral body fracture. (B) X-ray (lateral view) shows the extension of the construct to L1. (C) Intraoperative picture shows that only partial exposure of the existing construct was necessary to extend the fusion to L1.

An osteoporotic patient with a previous lumbar fusion (L2-S1) using the UNIMAX system who presented with an L2 vertebral body fracture after a fall. An extension of the fusion to L1 was done by the partial exposure of the cranial aspect of the previous construct and adding hardware for the additional level without removing any previous material (Figures 9A-9C).

## Results

The biomechanical testing showed the system was robust and met the requirements for FDA clearance. For the quasi-static torsion, the mean for the angular displacement, torsional stiffness, and torsional ultimate strength was 2.5 degrees (SD ± 0.8), 5.3 N-m/mm (SD ± 0.7), and 21.6 N-m (SD ± 4.4), respectively (Table 1).

**Table 1:** Results of biomechanical testing (bilateral construct quasi-static torsion) Abbreviations: Disp.; displacement, SD; standard deviation

Test#	Angular Disp. at 2% Offset Yield (deg)	Elastic Angular Disp. (deg)	Torsional Stiffness (N-m/mm)	Torsional Ultimate Strength (N-m)
1	2.2	1.2	4.4	30.1
2	1.5	0.5	5.8	22.1
3	4.1	3	5.2	21
4	1.9	0.9	6.5	14.9
5	2.6	1.6	4.9	20.5
Mean	2.5	1.4	5.3	21.6
SD	0.8	0.7	0.6	4.4

For the fatigue in axial compression, at an applied load of 1400 N, the closed ring construct showed a dynamic stiffness of 933.3 (N-m/m) and 5,000,000 (cycles to failure) with no construct failure (Table 2). The construct failed only when the applied load and bending moment were ≥ 1500 N and ≥ 60 (N.m.). For the construct quasi-static axial compression and bending, the mean for the elastic displacement, compressive yield bending moment, compressive bending stiffness, and compressive peak displacement was 16.3 mm (SD ± 3.6), 7545 N-mm (SD ± 1545), 465 N-mm/mm (SD ± 24.9), and 38.6 mm (SD ± 1.9), respectively (Table 3). 

**Table 2:** Results of biomechanical testing (bilateral construct quasi-static axial compression bending) Abbreviations: Comp.; compressive, Disp.; displacement, SD; standard deviation

Test #	Disp. at 2% Offset Yield (mm)	Elastic Disp. (mm)	Comp. Bending Yield Load (N)	Comp. Yield Bending Moment (N-mm)	Comp. Stiffness (N-mm)	Comp. Bending Stiffness (N-mm/mm)	Comp. Peak Disp. (mm)	Comp. Peak Force (N)
1	10.8	10	112	5040	11.2	504	37.7	225
2	19.2	18.4	177	7965	9.6	432.9	43.4	241
3	16.3	15.5	166	7470	10.7	481.9	40.8	237
4	21.7	20.9	209	9405	10	450	38	245
5	20	19.2	197	8865	10.3	461.7	39.5	237
Mean	17.6	16.8	172	7749	10.3	466	39.8	237
SD	4.2	4.2	37.5	1691	0.6	27.6	2.3	7.5

**Table 3:** Results of biomechanical testing (bilateral construct fatigue in axial compression)

Test#	Applied Load (N)	Applied Bending Moment (N.m)	Cycles to Failure	Failure Mode	Dynamic Stiffness (N-m/m)
1	1,800	72	99,761	1	789.6
2	1,600	64	241,205	2	1,014.0
3	1,500	60	1,141,000	3	1,192.1
4	1,400	56	5,000,000	NONE	933.3
5	1,200	48	5,000,000	NONE	703.3

## Discussion

Spinal instrumentation continues to evolve to achieve better surgical outcomes. Spinal fusion procedures are commonly used to treat various spinal pathologies, including degenerative conditions, deformity, trauma, infection, and tumors [1,4]. The existing pedicle screw-rod systems have increased the success rate and practicality of the fusion procedure. But it also has become apparent that there are limitations and failures in certain challenging situations [5-9].

The UNIMAX pedicle screw-plate system has unique features that make it a potentially valuable addition to the spine surgeon’s armamentarium. A plating system is inherently more stable than rods, especially in axial rotation [11]. One of the major advantages of the UNIMAX system is the creation of a rigid triangular construct at each spine level, a feature that is predicted to greatly increase the pullout resistance [12]. Vertical plates then interconnect the triangular constructs at each spinal level to increase the construct stability as it distributes the stress on both sides and across multiple levels. The flat sagittal plates can be contoured and stacked to provide the needed alignment and rigidity. Also, this design has a broad area of fixation between the flat vertical and flat horizontal plates, which reduces the cantilever and torsional motion and further increases the pull-out resistance provided by the screw threads and triangulation. By increasing the number of instrumented levels, the system increases rigidity and pullout resistance. In contrast, the screw-rod design suffers from an increase in the stress on the construct with a multilevel fixation and can lead to an increased failure rate [5-9].

A multilevel pedicle screw fixation requires a system that can accommodate different pedicle angles and inter-pedicular distances. The UNIMAX system achieves this by using the interlocking horizontal and vertical plates. The horizontal plates have a ball and socket type configuration to accommodate the varying pedicle screw angles, and the plates are available in different lengths with slots to accommodate any variability in the inter-pedicular/inter screw head distance. Another feature of this system is the ability to add additional vertical plates in a stacked and/or side-by-side configuration to provide additional posterior support to counteract both sagittal bending and torsional forces when needed. This feature can minimize the need for anterior column support, an especially useful advantage when dealing with osteoporosis and bone erosion from infection.

The UNIMAX system's versatility allows the correction of sagittal, coronal, or rotational deformity. To correct a sagittal deformity, the use of the extra-long square bolts on the horizontal plate will allow the addition of vertical plates to reduce the anteriorly displaced vertebra into proper alignment. For the correction of coronal or rotational deformity, an adequate posterior facetectomy is performed, allowing the triangular constructs at each level to transform each vertebra into an independent unit that can be brought into alignment by using the vertical plates as a scaffold.

The position of the vertical plates in the paramedian plane provides a larger bony surface area in the posterolateral area to contribute to the posterolateral boney fusion as compared to the traditional polyaxial screw-rod construct. This may enable a reduced need for simultaneous anterior interbody fusion or the use of fusion-promoting biologics with their inherent problems. The unique design and biomechanical characteristics of the UNIMAX system are predicted to increase the rate of bony fusion, which is important in all spinal fusion procedures and especially in clinical scenarios when robust fusion is of uttermost importance (e.g. osteoporosis or destruction of the vertebral body because of osteomyelitis or tumor invasion). Adjacent level disease is a known problem following instrumented spinal fusion. It sometimes requires the extension of a preexisting fusion, which can require the removal of the previously fused bone at multiple levels to allow for the release of the long rods. The UNIMAX system avoids this situation by simply adding sagittal extension plates that anchor the added level to the end of the existing construct.

Limitations

Despite the several advantages of the UNIMAX system, it does have several acknowledged limitations. First, the system requires an open posterior approach, which prevents it from being used with minimally invasive techniques. Also, this system in its current design can only be used in the thoracolumbar spine. A formal comparison of the long-term outcomes of the UNIMAX system in comparison to currently used screw-rod systems in a clinical setting is outside the scope of this report but we believe the theoretical advantages shown here warrant further study. Also, the metallic components of the system can affect the radiological evaluation of bony and neural structures (on CT or MRI).

## Conclusions

The pedicle screw and plate-based UNIMAX system has unique design features that seek to maximize the pullout strength through rigid triangulation at each spinal level. This system also increases the available surface area for posterolateral bony fusion, and it can correct some types of sagittal, coronal, and rotational deformities. It also allows the easy extension of an existing fusion construct to adjacent levels and can be incorporated intuitively into practice. It does require an open posterior approach that makes it incompatible with minimally invasive techniques. This system may have advantages over currently popular screw-rod systems in carefully selected situations requiring extra rigidity and high pull-out strength for complex reconstructions involving infection, tumor, poor bone quality, and revisions. We suggest that it warrants further testing in comparison to existing pedicle screw-rod systems.

## Figures and Tables

**Figure 10:**
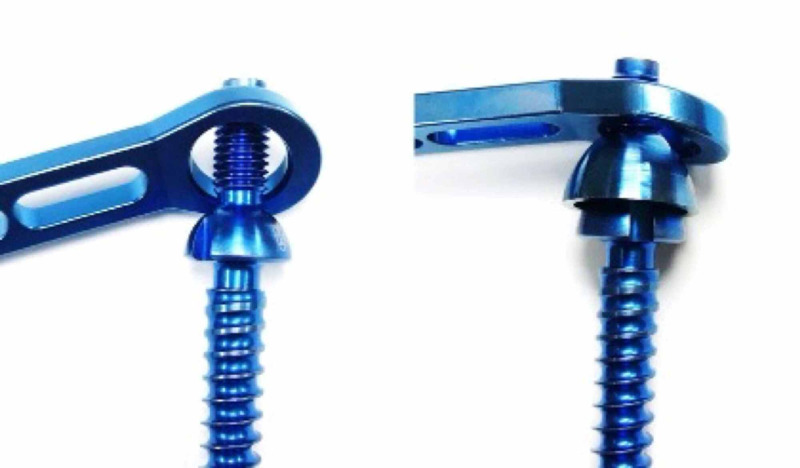
Pedicle screws

**Figure 11:**
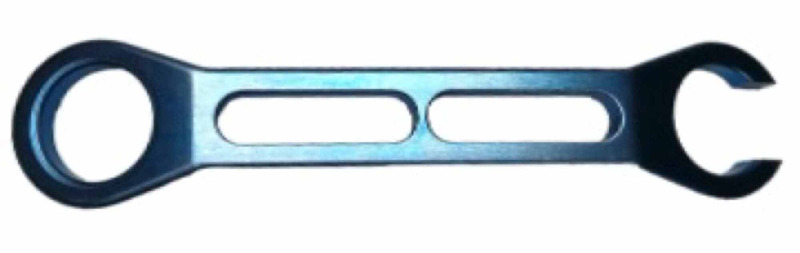
Horizontal plates

**Figure 12:**
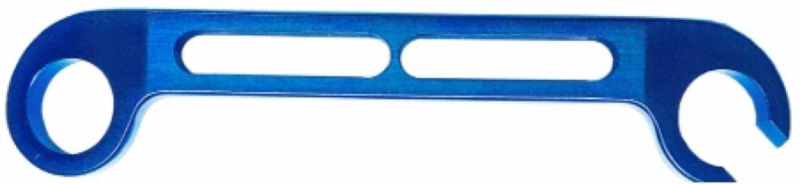
Horizontal plates

**Figure 13:**
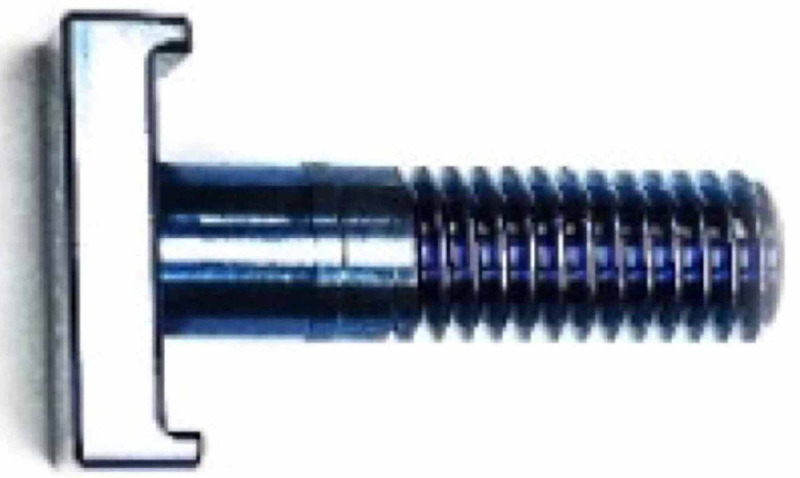
Square bolts (short)

**Figure 14:**
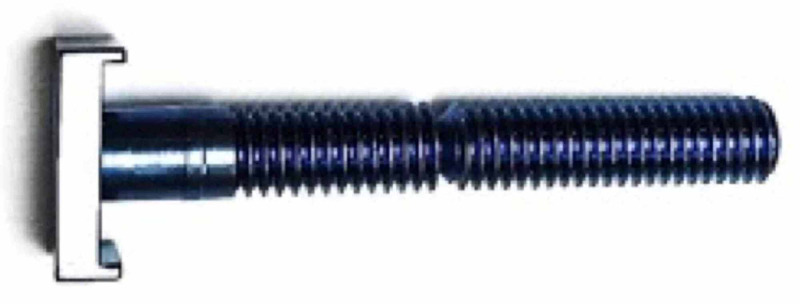
Square bolts (long)

**Figure 15:**
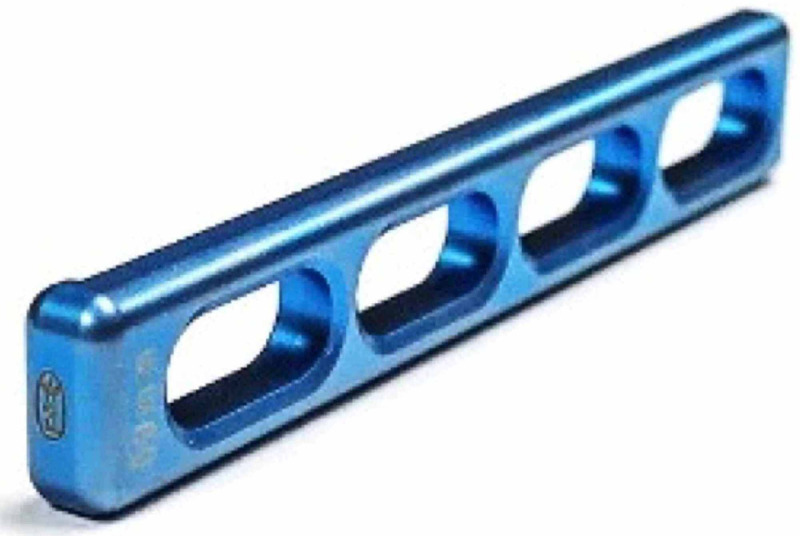
Vertical plate

**Figure 16:**
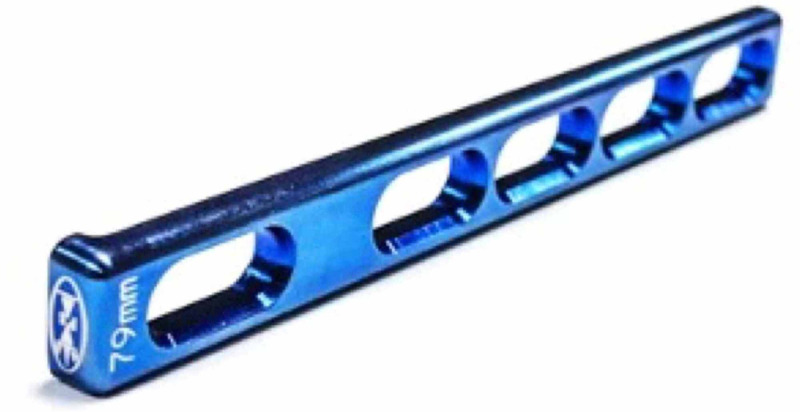
Vertical plate with offset

**Figure 17:**
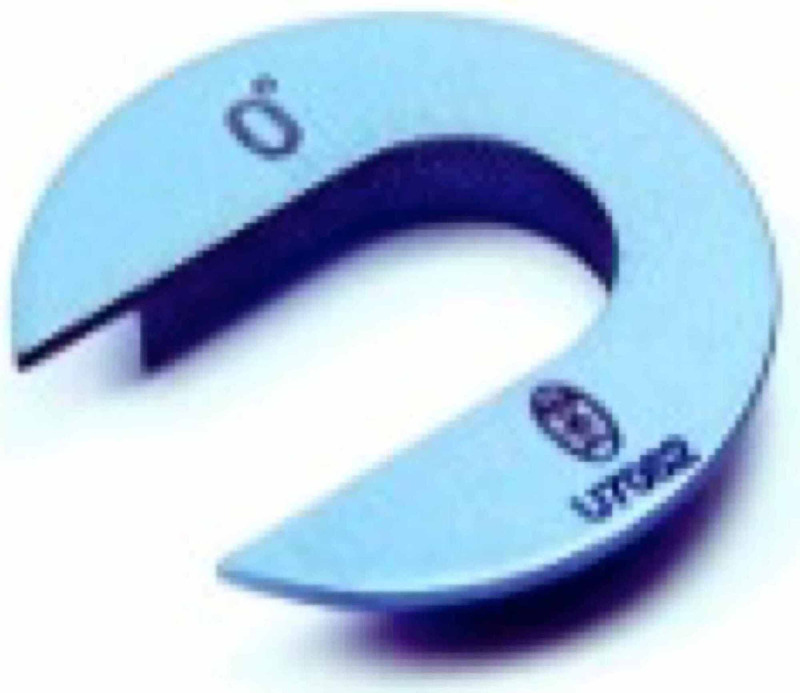
Flat washers

**Figure 18:**
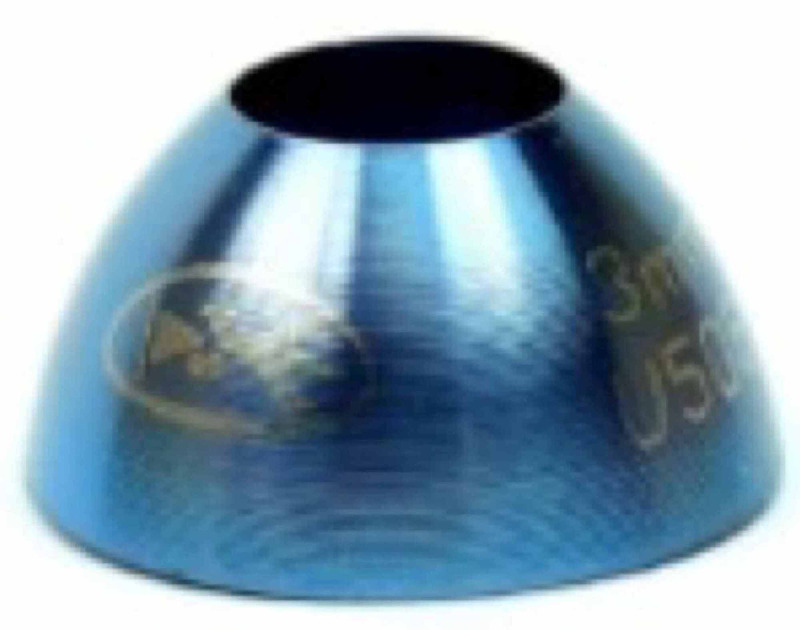
Cup washers

**Figure 19:**
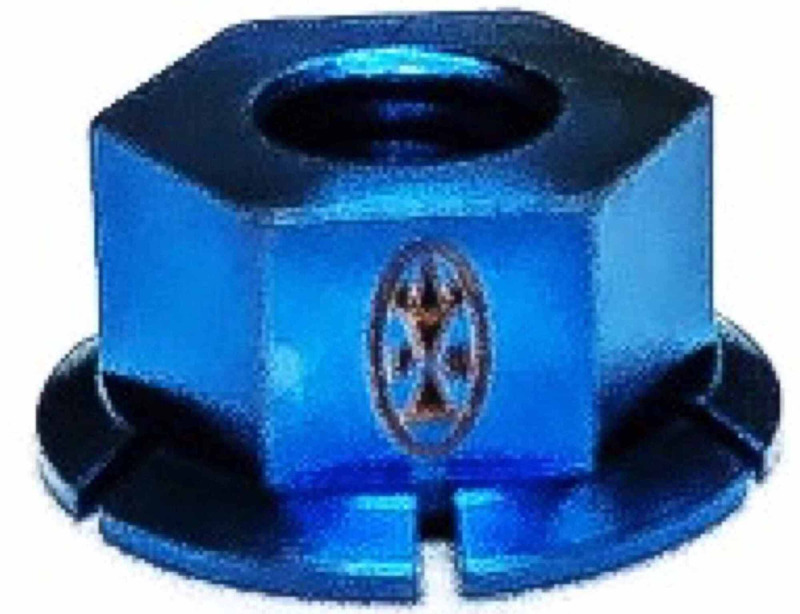
Large flange nuts

**Figure 20:**
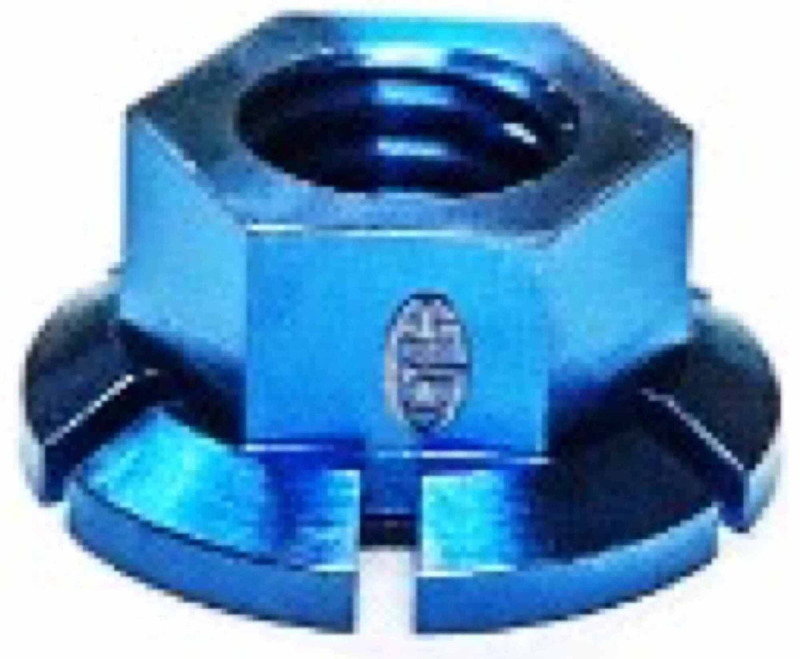
Small flange nuts

## Appendices

Description of the UNIMAX system components

1. Pedicle screws (Figure 10): These are simple with a hemispherical head without any multi-axis joint. The hemispherical top of the head creates a ball and socket relation with the concave undersurface of the horizontal plates.

2. Horizontal plates (length range 35-80 mm) (Figure 11): They have wider ends with circular holes to go over the screw heads. The circle is incomplete on one end to allow the plates to go down on the heads of the diverging screws. Between the ends, the horizontal plates have a left and right slot for accepting the slidable square bolts. Square bolts are brought into a straight line to accommodate vertical plates thus allowing multilevel fixation. The offset horizontal plates (Figure 12) are used to protect the upper non-fused facet joints.

3. Square bolts, these slide along with the slots on the horizontal plates on each side. The upward projecting shafts of the square bolts receive the vertical plates. The shafts are long enough to accommodate two vertical plates if needed (Figure 13). By sliding the square bolts of the multiple horizontal plates two straight lines are created to accept the vertical plates. There are special square bolts with a break-off-waist specially meant for use to reduce spondylolisthesis (Figure 14). These can also be used in other special needs like the reduction of rotoscoliosis. The “square” bolts have a rectangular base with ridges to grasp on to the undersurface of the horizontal plates. The shafts have a fine ridge around its waist that will snap into grooves on the inside of the slots on the horizontal plates to prevent the square bolt from falling during the surgery and to allow the sliding of the square bolts inside the slots.

4. Vertical plates, (length range 31-246mm) (Figures 15-16): They can accommodate most of the needs for fixation in multilevel degenerative spine disease. There are oval holes on the vertical plates to engage the square bolts. The so-called offset vertical plates have an extra-long bridge at one end to throw the holes to a different range to accommodate the square bolts in different situations.

5. Flat washers (Figure 17): They are U-shaped to reduce the large circular holes at the end of horizontal plates to a narrow slit to accept the large flange nuts that go down the top of the pedicle screws.

6. Cup washers (Figure 18): they go over the pedicle screw heads under the horizontal plates if it is necessary to raise the cross plate away from contact with the dura. They are necessary only in rare situations. Constant friction between the horizontal plates and the dura may potentially erode the dura over extended periods.

7. Large flange nuts (Figure 19): They go on to the pedicle screws to compress the horizontal plates with the flat washer and the concave under surface on to the hemispherical top of the pedicle screw head. This ball and socket arrangement will accommodate different pedicle screw angles.

8. Small flange nuts (Figure 20): They go on to the shaft of the square bolts to lock the vertical plates on to the horizontal plates.
